# Generic FPGA Pre-Processing Image Library for Industrial Vision Systems

**DOI:** 10.3390/s24186101

**Published:** 2024-09-20

**Authors:** Diogo Ferreira, Filipe Moutinho, João P. Matos-Carvalho, Magno Guedes, Pedro Deusdado

**Affiliations:** 1 INTROSYS SA, 2950-805 Quinta do Anjo, Portugal; magno.guedes@introsys.eu (M.G.); pedro.deusdado@introsys.eu (P.D.); 2 NOVA School of Science and Technology, NOVA University Lisbon, 2829-516 Caparica, Portugal; fcm@fct.unl.pt; 3 Center of Technology and Systems (UNINOVA-CTS) and Associated Lab of Intelligent Systems (LASI), 2829-516 Caparica, Portugal; joao.matos.carvalho@ulusofona.pt; 4 COPELABS, Centro Universitário de Lisboa, Universidade Lusófona, 1749-024 Lisbon, Portugal

**Keywords:** FPGA, GPU, pre-processing image library, industrial vision systems

## Abstract

Currently, there is a demand for an increase in the diversity and quality of new products reaching the consumer market. This fact imposes new challenges for different industrial sectors, including processes that integrate machine vision. Hardware acceleration and improvements in processing efficiency are becoming crucial for vision-based algorithms to follow the complexity growth of future industrial systems. This article presents a generic library of pre-processing filters for execution in field-programmable gate arrays (FPGAs) to reduce the overall image processing time in vision systems. An experimental setup based on the Zybo Z7 Pcam 5C Demo project was developed and used to validate the filters described in VHDL (VHSIC hardware description language). Finally, a comparison of the execution times using GPU and CPU platforms was performed as well as an evaluation of the integration of the current work in an industrial application. The results showed a decrease in the pre-processing time from milliseconds to nanoseconds when using FPGAs.

## 1. Introduction

The constant technological evolution of recent decades has resulted in the emergence of increasingly efficient solutions in diverse areas. The industrial sector was one of the sectors most positively affected by this evolution. The high level of industrialization worldwide implies increasing competition between companies to achieve success, where product quality is currently a decisive factor in consumer satisfaction.

Tasks are performed by specialized personnel, who, despite being experts in the field, are prone to errors due to fatigue or the complexity of the tasks. To reduce these errors, the industrial sector has promoted higher product quality by introducing automated systems, where tasks are now performed repeatedly by machines assisting or replacing humans, reducing the occurrence of errors and improving safety, such as in ref. [1,2].

Currently, one of the areas where automation is predominant is industrial vision. Vision systems are characterized by three sequential phases. The process begins with the acquisition of an image or video; then, it undergoes processing, where the relevant features are extracted, and finally, the system makes decisions according to the analysis of the results obtained. These can be applied to object detection and classification as well as to quality control applications. The main process of this type of system is image processing. This usually runs on CPU, but the need to obtain better-quality and faster products has resulted in an increase in the complexity of this type of system, requiring the integration of more hardware for improvements in speed and efficiency. Graphic processing units (GPUs) and FPGAs have been proposed for real-time detection [3,4,5,6].

An objective of this work is the development of a generic library of pre-processing image filters in FPGAs to reduce the processing time in vision systems, and consequently, to reduce their cycle time. Additionally, an experimental setup was developed, which was used to validate the proposed methods and compare their execution times with two other platforms (CPU and GPU).

In this article, a state-of-the-art section is first presented (Section 2), in which references about the three platforms under study (FPGA, CPU, and GPU) are analyzed as well as existing industrial vision applications. Then, in Section 3, the proposed library is presented. In Section 4, the experimental setup architecture is presented, where it is possible to have a perception of all the modules of the project. The experimental results section presents the results obtained regarding the processing times of the filters on the three platforms as well as the integration of the project in a real industrial application (Section 5). Finally, the results are discussed in Section 6, and the conclusions are given in Section 7.

## 2. Related Work

The need for faster processing requires the use of platforms with hardware acceleration capabilities (FPGA, CPU, and GPU). In ref. [7], a study of the existing CPU, GPU, and FPGA solutions was performed, where the high level of portability and parallelism, flexible behavior throughout the implementation, and a potentially higher data processing speed are given as some of the features of FPGAs that make them useful for applications with significant data quantity and processing needs. However, their use makes the task of developing projects more complex. Currently, FPGAs are used in diverse areas and applications, including noise cancellation [8], embedded intelligence [9], and IoT [10].

In the area of image processing, several studies have been performed on these three platforms in different use cases, including vision systems [11], the application of the convolution of two masks and sum of absolute differences algorithm [12], and execution algorithms using OpenCL [13]. In ref. [14], a comparison was made between FPGA/GPU platforms, where their features were analyzed, while ref. [15] focused on a similar analysis applied to FPGA and CPU platforms.

Hardware can be developed using several approaches, including HDL languages [16], high-level synthesis [17], or OpenCL [18]. Image processing can be performed on this platform using these three methods. In refs. [19,20], the authors used VHDL to implement filters. In refs. [21,22], high-level synthesis was used, and in ref. [23], the authors used OpenCL. Based on these references, it can be stated that the use of hardware description languages favors a faster, more flexible, and more efficient solution. However, the development time is longer when compared to the other two solutions.

Currently, there are vision systems that use FPGAs in their constitution [24,25,26]. Object detection [27,28] and classification [29] are two areas where these platforms contribute to image pre-processing. These articles demonstrate the important role of this platform in the vision systems to which it was applied, favoring their performance. Despite the advantages of the use of FPGAs for image processing when using HDL, they are not widely used due to the lack of freely available image processing libraries.

## 3. Proposed Library

This section presents the proposed pre-processing image library, which is composed of 10 widely used filters designed and described in VHDL. The filter’s input is an AXI-Stream protocol bus [30] with the information of the current pixel value (RGB, gray, or binary), its position in the frame, and a clock signal for synchronization, whereas the output is an AXI-Stream protocol bus with the values of the processed pixels.

### 3.1. RGB/Gray

The RGB/Gray filter was designed to convert three component pixels (RGB) to one (gray) [31]. To accomplish this, a mean was applied to the three component values; however, in VHDL, the division operator can only be used if the denominator corresponds to a power of 2. Therefore, the following rounding equation was used (Equation (1)).

(1) GrayValue=(R+G+B)×171÷512

### 3.2. RGB/YCbCr

The YCbCr image format can be useful in some specific situations, such as face recognition [32,33]. To convert RGB pixels to YCbCr, the following rounding Equation (2) was used.

(2) $$\begin{array}{c} Y=(4\times R+8\times G+2\times B+256)÷16\\ Cb=(-2\times R-5\times G+7\times B+2048)÷16\\ Cr=(7\times R-6\times G-B+2048)÷16 \end{array}$$

### 3.3. Inverse

The inverse filter modifies the RGB value of the current pixel to its complement. If the original pixel is dark, the processed one will be bright and vice versa. This module was developed using Equation (3).

(3) Inverse(R,G,B)=255−Component(R,G,B)

### 3.4. Brightness

To modify the brightness of RGB pixels, a reference value is added to the original pixel value. If the reference is positive, the output pixel will be brighter; otherwise, it will be darker. Equation (4) was used to develop the module with a condition to limit the output values between 0 and 255.

(4) Bright(R,G,B)=Component(R,G,B)+Reference

### 3.5. Binary

A grayscale-to-binary conversion reduces the image complexity. This module has the following behavior: if the original pixel value is greater than a predefined threshold, the output is white (255); otherwise, it is black (zero).

### 3.6. Convolution Mask

About the filters already presented, the result of their application depends only and exclusively on the pixel value to be processed. The methods described below are more complex, since the result of their application depends on the pixel to be processed, its neighbors, and a convolution mask. The mask chosen to implement this type of filter has a 3 × 3 dimension.

Due to the high resolution of the image captured by the camera (1920 × 1080 pixels), the region of interest was delimited to simplify the algorithm implementation and increase its speed.

**Figure 1:**
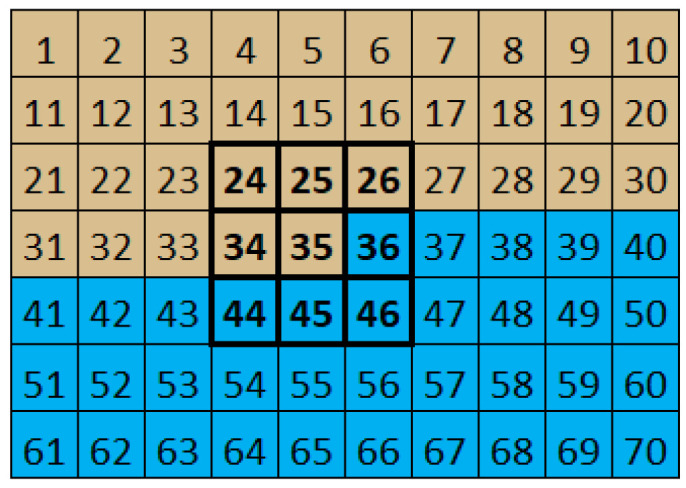
First application scenario (the pixels received are represented in brown and those that have not yet been received are in blue).

One of the most important aspects in the development of the convolution mask algorithm is the knowledge of the position to be processed. Sequential pixel reception, from left to right and from top to bottom, prevents the filter from being applied immediately after receiving the central pixel of the mask. This is because this operation depends on neighbors that have not yet been received. Figure 1 shows a situation where it is not possible to process the pixel with the value 35 because there are neighbors that have not yet been received.

**Figure 2:**
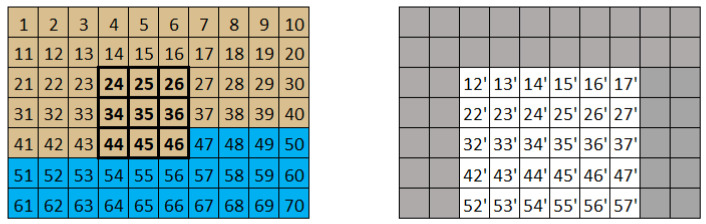
Convolution mask algorithm application (the original image is shown on the left, and the processed image is shown on the right with the gray areas representing the unprocessed borders).

The algorithm works as follows: when the current pixel is in the processing region, the convolution mask moves so that the current position is always in the bottom right corner, allowing access to the values necessary for applying the method. Under these conditions, the central pixel is processed and placed in the resulting image with an offset of one row and one column from its original position. As a result, the resulting image loses its borders and is displayed with the explained offset. Figure 2 illustrates the application of this method, showing the original image on the left, where the filter can be applied to the pixel with a value of 35, and the resulting image on the right side.

After developing the algorithm for the 3 × 3 convolution mask, modules were implemented based on the mentioned procedure. For this purpose, registers were used to control the filter application on each pixel (Table 1).

The “Resolution_x” and “Resolution_y” registers represent the dimensions of each frame (1920 × 1080 pixels). The registers “Count_x” and “Count_y” indicate the current position (horizontal and vertical) of the current pixel in the frame, as the exact location of the pixel to be processed is required. Finally, to access the nine values of the convolution mask, it was necessary to store the two rows preceding the current one (“Buffer_1” and “Buffer_2”) as well as the two preceding pixels (“Buffer_3”). Since the last two columns of each frame are not processed, the first two vectors have a dimension of 1918, while the third buffer has only two positions.

**Figure 3:**
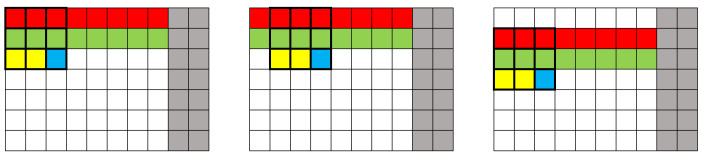
Representation of the application of the convolution mask in 3 situations (in red is represented “Buffer_1”, in green “Buffer_2”, in yellow “Buffer_3”, the current pixel in blue and the two columns that are not processed in gray).

Figure 3 shows three different situations: the filter applied to the second pixel of the second row (left image), the third pixel of the second row (middle image), and the second pixel of the third row (right image). From the analysis of these images, it can be concluded that the position of the buffers in the frame must be changed as the pixel values are received to guarantee the correct application of the method.

In order to process frames by the mentioned algorithm, it is necessary to make some checks about the exact position of the pixel. If its location is in the neglected region, the filter is not applied, and the buffers are updated; otherwise, the filter is applied because the pixel is located in the region of interest. The sequential procedures for checking the location of the current pixel in the frame are presented in Algorithm 1.
**Algorithm 1** Convolution mask algorithm  1:Resolution_x←1920  2:Resolution_y←1080  3:Count_x←0  4:Count_y←0  5:Buffer_1[Resolution_x−2]  6:Buffer_2[Resolution_x−2]  7:Buffer_3[2]  8:**if** TUSER **then**  9:    Pixel_out←Pixel_in10:    Buffer_1[Count_x]←Pixel_in11:    Count_x←Count_x+112:**else if** Count_y = 0 **then**13:    Pixel_out←Pixel_in14:    Buffer_Data(Buffer_1,Count_x,Count_y)15:**else if** Count_y = 1 **then**16:    Pixel_out←Pixel_in17:    Buffer_Data(Buffer_2,Count_x,Count_y)18:**else if** Count_x = 0 **then**19:    Pixel_out←Pixel_in20:    Buffer_3[0]←Pixel_in21:    Count_x←Count_x+122:**else if** Count_x = 1 **then**23:    Pixel_out←Pixel_in24:    Buffer_3[1]←Pixel_in25:    Count_x←Count_x+126:**else if** Count_x = Resolution_x−1 **then**27:    Pixel_out←Pixel_in28:    Buffer_1[Count_x−2]←Buffer_2[Count_x−2]29:    Buffer_2[Count_x−2]←Buffer_3[0]30:    Buffer_3[0]←Buffer_3[1]31:    Count_x←Count_x+132:**else if** TLAST **then**33:    Pixel_out←Pixel_in34:    Buffer_1[Count_x−2]←Buffer_2[Count_x−2]35:    Buffer_2[Count_x−2]←Buffer_3[0]36:    Count_x←037:    Count_y←Count_y+138:**else**39:    Pixel_out←Processed_pixel40:    Buffer_1[Count_x−2]←Buffer_2[Count_x−2]41:    Buffer_2[Count_x−2]←Buffer_3[0]42:    Buffer_3[0]←Buffer_3[1]43:    Buffer_3[1]←Pixel_in44:    Count_x←Count_x+145:**end if**46:**function** Buffer_Data(Buffer, Count_x, Count_y)47:    **if** Count_x≠Resolution_x−1 **then**48:        **if** TLAST **then**49:           Count_x←050:           Count_y←Count_y+151:        **else**52:           Buffer[Count_x]←Pixel_in53:           Count_x←Count_x+154:        **end if**55:    **else**56:        Count_x←Count_x+157:    **end if**58:**end function**

The following subsections present a set of algorithms based on the described convolution mask.

### 3.7. Sobel

Edge detection techniques, such as the Sobel filter, play a crucial role in image processing [34,35]. The developed Sobel module implements Equations (5)–(7), which are supported by a 3×3 convolution mask. Note that “a”, “b”, and “c”; “d”, “e”, and “f”; and “g”, “h”, and “i” are the three rows of the mask.

(5) Sx(R,G,B)=(a+2×d+g)−(c+2×f+i)

(6) Sy(R,G,B)=(a+2×b+c)−(g+2×h+i)

(7) $$S\left(R,G,B\right)=\left|Sx\right|+\left|Sy\right|$$

### 3.8. Mean

The mean filter is a noise reduction method designed to process RGB pixels [36]. Its output value is the result of rounding the arithmetic mean including all nine values contained in the convolution mask (3 × 3). The developed filter implements Equation (8).

(8) $$Mean\left(R,G,B\right)=\frac{\sum M(i,j)*57}{512}$$

### 3.9. Gaussian Filter

**Figure 4:**
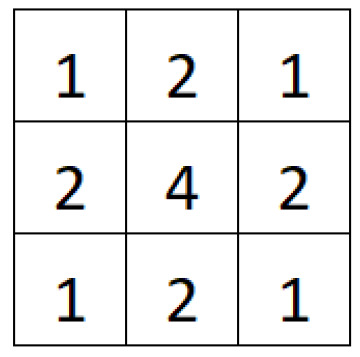
Convolution mask used to develop the Gaussian filter module.

Another noise reduction filter is the Gaussian filter. The method can be explained in three steps: first of all, each position of the convolution mask (Figure 4) is multiplied by the corresponding pixel values in the image; then, all nine results are summed, and finally, the final value is divided by 16.

### 3.10. Erosion

The erosion filter aims to reduce noise on binary images. To develop this method, a convolution mask composed of zeros was compared to the corresponding pixels. The output is black (zero) if all the pixel values are equal to all mask values; otherwise, the output pixel is white (255).

### 3.11. Dilation

The dilation filter is also applied to binary images but with a different goal: instead of reducing noise, it adds information to the image. Like the erosion filter, a convolution mask of zeros is compared to the corresponding pixels, but the output is black (zero) if at least one pixel value is equal to the corresponding mask value; otherwise, the output pixel is white (255).

## 4. Experimental Setup

The following experimental setup was used to validate the developed pre-processing methods and to compare three different approaches: pre-processing the image in FPGAs, CPUs, or GPUs. In all cases, the image acquisition is performed by the FPGA. The image processing stage can take place in the three platforms, so the original or processed data (depending on where the pre-processing filters are applied) is transmitted from the FPGA to the CPU via User Datagram Protocol (UDP).

A pre-processing image vision system is composed of three sequential stages: image acquisition, image processing, and data transmission for feature extraction. To develop this project, a hardware platform with these features was needed, so the Zybo Z7-20 [37] was chosen because of its connectivity peripherals, video capabilities, and direct integration with the Zybo Z7 Pcam 5C demo from Digilent (Pullman, WA, USA) [38]. The Zybo Z7-20 has the following specifications: ZYNQ processor (667 MHz dual-core Cortex-A9 processor; DDR3L memory controller with 8 DMA channels and 4 high-performance AXI3 slave ports; and high-bandwidth peripheral controllers: 1G Ethernet, USB 2.0, and secure digital input output), memory (1 GB DDR3L with 32-bit bus @ 533 MHz), Ethernet (Gigabit Ethernet PHY5), 3200 Look-up Tables, 106400 Flip-Flops, and 630 KB for Block RAM.

### 4.1. Architecture

**Figure 5:**
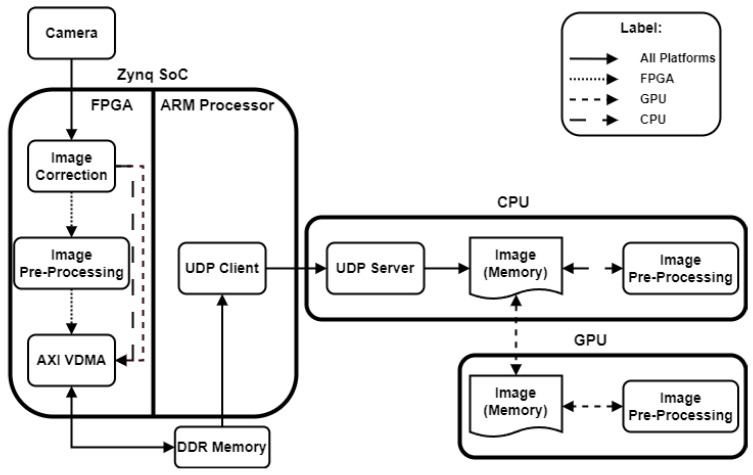
Pre-processing module location in each architecture.

Three architectures have been developed, each executing the pre-processing block on one of the platforms mentioned above. Figure 5 illustrates the placement of each pre-processing module within the respective architectures. To clarify the execution responsibility of each platform, four distinct line styles are used: solid lines represent blocks executed by all platforms, dotted lines indicate FPGA modules, short dashed lines denote GPU blocks, and long dashed lines correspond to CPU blocks.

Considering the case where the pre-processing is completed in the FPGA, the video captured by the camera is filtered pixel by pixel in real time, and then the processed frame is sent to the CPU to be stored in memory. In the other two cases (CPU and GPU), the image captured by the camera is received and stored in the CPU memory without any type of processing, and then filters are applied to it in the CPU or GPU. In conclusion, the pre-processing block uses a different approach depending on the platform where it is executed: in the FPGA, the module receives, processes, and returns a pixel in real time, which is unlike the other two platforms where the image pixels are read from the memory, processed, and updated.

### 4.2. Zybo Z7 Pcam 5C Demo

The Zybo Z7 Pcam 5C design receives real-time data from the camera (Pcam 5C) via MIPI protocol and streams it out through the HDMI TX port. Zybo Z7-20 includes a UART module used by the user to configure some image sensor definitions (resolution, image format, and gamma correction factor value) and hardware IP cores.

The circuit is based on the AXI4-Stream protocol [30] to transmit the received pixel values to the HDMI TX port. This protocol is characterized by master/slave communication and is composed of several signals to ensure the correct behavior between modules. The TDATA signal has 24 bits and represents the RGB pixel value (each component has 8 bits). The binary signals TVALID and TREADY are responsible for the correct communication between two modules; the first one indicates if a master has data to transmit, while the second one indicates if one slave is ready to receive information from the corresponding master. The TUSER signal indicates if the current pixel is in the first position of the frame (top left corner), and the TLAST signal indicates if the current pixel is in the last row of the frame.

**Figure 6:**
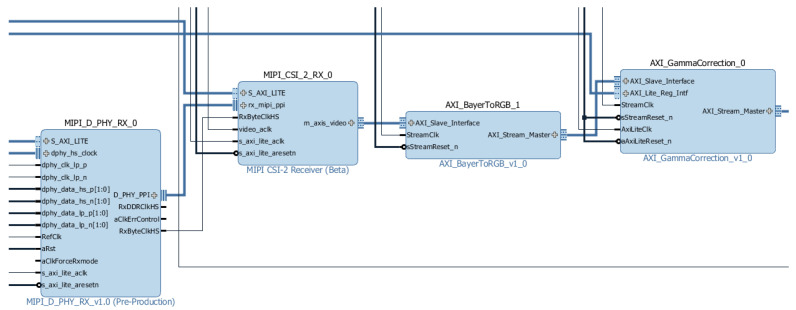
Image acquisition and gamma correction modules.

**Figure 7:**
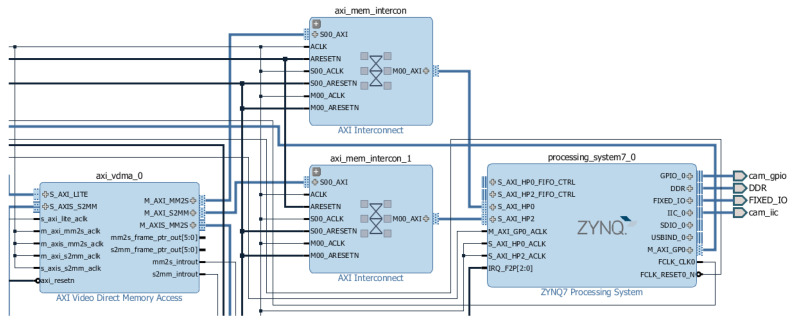
VDMA and Zynq processing modules.

The hardware part of the demo can be divided into four sections: image acquisition, gamma correction, connection to the Zynq processing system, and data streaming via HDMI. Image acquisition is handled by two modules that convert the received RAW data to AXI4-Stream buses. The gamma correction stage is characterized by the conversion from of RAW RGB to RGB pixels with 24 bits each (values ranging from 0 to 255 per component) as well as the application of a gamma correction factor (1/1.8) that influences the input data brightness (Figure 6). The connection with the Zynq processing system is made by a VDMA IP that has two main functions: to store three Full HD frames (1920 × 1080p) on a DDR memory to be accessed by the Zynq processing system (circular buffer) and to transmit the AXI4-Stream bus to the HDMI section (Figure 7). The last stage is responsible for streaming the data out through HDMI; this process is performed by four modules that generate HDMI control signals, synchronize, and stream the output value (RGB pixel).

To integrate this demo into the current project, some modifications were made. Pre-processing filters were added between the gamma correction stage and the VDMA module to process the input video. Additionally, instead of storing three frames in the DDR memory, ten frames were stored to ensure the correct transmission of frames to the CPU.

### 4.3. Frame Transmission

To validate the developed filters on the FPGA, as well as to enable the application of CPU/GPU pre-processing algorithms, and finally to allow the integration into a real industrial vision system, a frame transmission/reception process from the FPGA to CPU based on Ethernet protocol (UDP) was implemented.

The process of transmitting frames to the CPU was implemented in C language using the Vivado SDK (version: 2016.4) software and then integrated into the demo that served as the project’s base architecture. Since the VDMA IP core stores frames in DDR memory (accessed by the Zynq processing system), a pointer variable was used to access all positions and send its values to the CPU. Given that each frame has a resolution of 1920 × 1080 pixels (2,073,600 bytes considering a grayscale image) and that each row of the frame was divided into three packets of 640 bytes, a total of 3240 UDP packets were required to transmit a single frame from the FPGA to the CPU.

The data buffer to be sent is 1440 bytes long. The first two positions contain the packet number (ranging from 0 to 3239), the next 640 positions contain the pixel values to be transmitted, and the remaining positions are unused.

With the frame transmission implemented, a C# 4.0 application was developed to receive the frames on the CPU. The image reception process can be described as follows: first, an image and the corresponding pointer are created; then, the UDP server is started, which receives information in real time. As the UDP protocol does not guarantee the correct ordering of the sent packets, a check is performed to disregard out-of-order packets. When a packet is correctly received, the pixel values are placed in the image created at the beginning of the process, and the pointer is incremented until the image is complete. At the end of the frame, the pointer is reset, and the same process is repeated for the next frame.

## 5. Experimental Results

After developing and integrating the filters into the Zybo Z7 Pcam 5C demo, an industrial vision system was created. Processing times were collected and compared to CPU- and GPU-based implementations.

### 5.1. Filters Results

**Figure 8:**
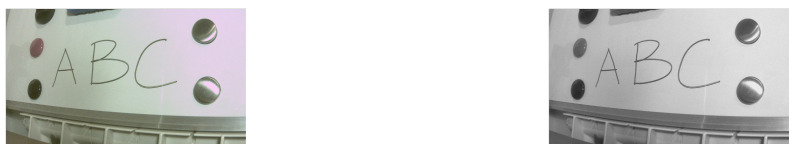
Application of the RGB/Gray conversion (original image on the **left** and the processed image on the **right**).

**Figure 9:**
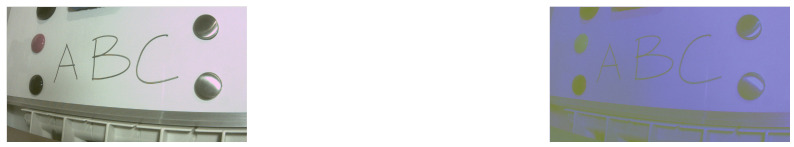
Application of the RGB/YCbCr conversion (original image on the **left** and the processed image on the **right**).

**Figure 10:**
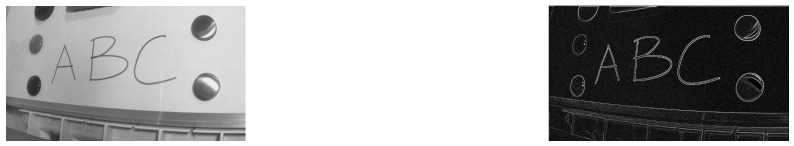
Application of the Sobel filter (original image on the **left** and the processed image on the **right**).

This subsection presents some results from the application of the developed filters (Figure 8, Figure 9 and Figure 10). The original image was captured by the PCAM 5C camera, processed by the developed modules implemented on the FPGA, and then transmitted and stored in the CPU memory.

### 5.2. Filters Processing Time

The processing time of each filter was measured on three different platforms: FPGA, GPU, and CPU. For the FPGA, the execution time was measured by the clock signal frequency and the number of cycles required to apply the filter (Equation (9)). Table 2 shows the measured times for all developed methods.

(9) $$FPGA\;Processing\;Time=\frac{Clock\;Cycles\;per\;Filter}{Clock\;Frequency}$$

For CPU and GPU processing, the same 10 filters were implemented through the equations mentioned in the previous section, which were followed by the measure of the full application period of the algorithms, i.e., the time per frame. This was used to calculate the processing time of a pixel (Equation (10)). Note that the CPU implementation was based on the C# 4.0 language using the EmguCV library, and the GPU implementation made done using Numba Python GPU (version 0.57.0) in a Ubuntu operating system.

(10) $$GPU/CPU\;Processing\;Time=\frac{Total\;Time}{Frame\;Resolution}$$

To obtain more accurate results, 30 samples were measured, and the mean time and the fastest time were calculated. The results related to GPU processing times are presented in Table 3, and the results related to CPU processing times are presented in Table 4.

Throughout the FPGA implementation process, detailed resource utilization, including Look-Up Tables (LUTs), Flip-Flops (FFs), Look-Up Table Random Access Memory (LUTRAM), and Block Random Access Memory (BRAM), was carefully examined to measure the efficiency of the design. The usage of these key resources was closely monitored to ensure the optimal performance and effective use of the FPGA architecture. Table 5 summarizes the results and provides a quantitative breakdown of resource consumption where each BRAM unit can store 36 Kb.

### 5.3. Industrial Application

After developing an image pre-processing system on the FPGA, it was integrated into an innovation project (CheckMate) for industrial vision-based quality inspection, which was carried out by Introsys [39]. The project architecture includes a guided platform with a collaborative robot equipped with a gripper to carry out the quality inspection of several specific characteristics of the car. One of the use cases is the visual evaluation of the displacement and rotation of the dashboard buttons after the assembly process. This task is time-critical for its viability in the final assembly line of car manufacturers, where reducing the processing time can reduce the number of quality control stations and thus significantly reduce costs.

**Figure 11:**
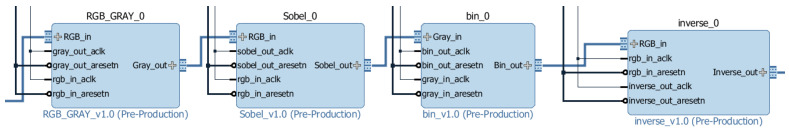
The filters used in the developed industrial application.

To identify the defects, a sequence of filters was developed to highlight and detect edges. The filters used in sequence were RGB/Gray, Sobel, Binary, and Inverse, as presented in Figure 11. The first one, which receives data from the AXI gamma correction module, converts an image with three channels into a single channel. The second filter highlights the edges in the grayscale images. The following module converts the grayscale image to black and white (where edges are highlighted in white). Finally, to obtain black edges, the inverse filter was used. The output of the inverse filter is connected to the AXI VDMA module. It is important to note that to implement this sequence, it was required to reduce the clock frequency from 150 to 100 MHz to ensure the required signal propagation delays.

**Figure 12:**
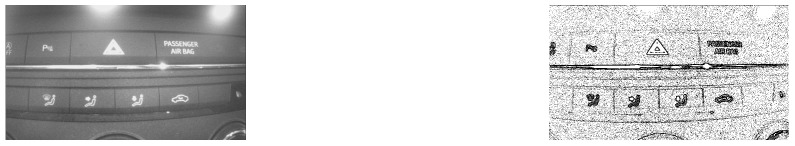
An image resulting from the integration of the present project in a real industrial vision system (original image on the **left** and the processed image on the FPGA on the **right**).

To compare the performance of the FPGA, GPU and CPU, the pre-processing was performed on the FPGA (in VHDL); CPU and GPU (direct implementation in C# of the algorithms designed in VHDL); CPU (using Halcon 18.11 [40]); and CPU (using OpenCV). An image resulting from the FPGA implementation is shown in Figure 12, while the processing times are presented in Table 6. Note that the execution time of the RGB to gray conversion is not included in Table 6. This is because the RGB to gray conversion was only implemented in the FPGA to ensure that the same frame was processed in the CPU and the GPU.

## 6. Discussion

This section presents a discussion related to the execution times of each filter on the three platforms under study as well as their processing times when integrated into a real application.

### 6.1. Developed Algorithms

To understand the comparisons between the three platforms, it is necessary to keep in mind that the application of filters by the FPGA takes place in real time; i.e., as soon as the pixel values are received, they are immediately processed. On the other hand, in the CPU/GPU, the processing was completed by applying the filter to an image already stored in memory. Regarding the GPU, it should be noted that the total processing time of an image is equal to the sum of the transmitting time of the two images between the CPU and the GPU (original and processed) in addition to the time required to apply the filter. Specifically, it takes 3.3 ms to send the original image to the GPU and 5.5 ms to send the processed image back to the CPU. Thus, 8.8 ms is required just for image transmission between the two platforms before applying a filter on the GPU.

The computer used for GPU processing has the following specifications: Intel i7-8750H CPU @ 2.20GHz*12 with 12 cores (Intel, Santa Clara, CA, USA), 16 GB memory RAM, and NVIDIA GeForce GXT 1060 (NVIDIA, Santa Clara, CA, USA), on the Ubuntu 20.04 operating system.

When comparing the performance of the FPGA and CPU in terms of time per pixel, the CPU generally gives better results except for the mean processing time of the uniform mean filter. However, when considering the entire frame and the fact that the FPGA applies filters in real time, the processing delay for a frame on the FPGA is proportional to the delay for a single pixel. For example, in the RGB/Gray method, applying the filter delays each pixel by 1 clock cycle (10 ns). As a result, the entire frame is delayed by only 1 clock cycle because it is processed immediately after the pixel is received. Therefore, when comparing frame processing times, the FPGA achieves significantly better results with delays on the order of nanoseconds compared to the milliseconds required by the CPU. This demonstrates that the FPGA offers superior real-time processing performance due to its shorter delay period. The computer (LAPTOP-C08RVMIO) used to develop the CPU algorithms has the following specifications: Windows 10 Home, Intel(R) Core(TM) processor i7-8550U CPU @ 1.80 GHz 2.00 GHz (4 cores), 16 GB of RAM, and 256 GB of SSD memory.

When comparing the performance between the FPGA and the GPU for image pre-processing, the FPGA only delays each frame by a maximum of 6 clock cycles (60 ns) at a clock frequency of 100 MHz (Sobel filter), while the GPU implementation requires approximately 8.8 ms to process an image regardless of the filter used. From these results, it is possible to conclude that in this specific situation, FPGA image pre-processing is more efficient than GPU image pre-processing.

Considering the results obtained between the CPU and GPU, it is possible to verify that the GPU filter execution per frame and pixel is much faster than that of the CPU; however, the sending and receiving time of images between the two platforms must be considered (8.8 ms). Taking this into account and considering the processing of a full frame, it is possible to affirm that all methods except binary and inverse are executed faster on the GPU.

The power consumption of the used platforms (FPGA, CPU, and GPU) is shown in Table 7. The results clearly show that the FPGA is the most power-efficient option, consuming between 1 and 5 watts. In contrast, the GPU power consumption is significantly higher, ranging from 10 to 125 watts, while CPU power consumption is relatively moderate, but still higher than FPGA, at approximately 15 watts. In conclusion, this analysis highlights the superior power efficiency of the FPGA when compared to the other platforms.

To conclude, in terms of resource usage, and because the language used is VHDL, the methods developed are adaptable and can be implemented in any FPGA that meets the required resource specifications. This flexibility ensures compatibility across different FPGA architectures, allowing versatile and efficient use in different hardware environments.

### 6.2. CheckMate Integration

Regarding the integration into the Checkmate system, the processing time was also measured on the three platforms: FPGA, CPU (Halcon), CPU (OpenCV), and through the equations developed in VHDL (CPU/GPU). In the FPGA, each frame is delayed by 8 clock cycles, i.e., 80 ns. Considering that the CPU processing is not performed in real time, the time per frame obtained by using the Halcon tool is 30.2389 ms, while that obtained by using the OpenCV library functions is 37.2415 ms, and that by implementing the equations implemented in the FPGA is 115.3149 ms. Note that the methods of the Halcon tool and OpenCV library process the borders, which does not happen in the FPGA implementation. Regarding the times measured on the GPU and considering the 8.8 ms of image transmission between the CPU and GPU, it is possible to verify that the execution time on this platform was approximately 9 ms.

The results show that the CPU and GPU implementations are more efficient when comparing the processing time per pixel. However, when considering the real-time processing per frame, the FPGA achieves better results. In this scenario, each frame is delayed by only 8 clock cycles (80 ns) for processing, while using the Halcon tool (fastest result on CPU) requires 30.2389 ms, and the GPU requires 9 ms for sequence application. It can be concluded that image pre-processing on an FPGA offers clear advantages in terms of processing time compared to CPU and GPU implementations.

## 7. Conclusions

In this work, a pre-processing image library of 10 filters has been developed in VHDL for FPGAs, which despite their known advantages are not widely used in industrial vision systems. To apply and validate the developed filters, an experimental setup has been developed based on the Zybo Z7 Pcam 5C demo project, which includes the implementation of a frame transmission over Ethernet.

A real-time image pre-processing system was successfully developed, allowing filter validation and integration into industrial vision applications. The results were validated by comparing images processed on an FPGA, CPU, and GPU. The processing times measured on three platforms show that FPGA execution is faster than both CPU and GPU. FPGA processing times are on the order of nanoseconds, while the other platforms have times in the order of milliseconds. It can also be concluded that the use of the GPU is only advantageous when the number of sequential filters is high. Under these circumstances, the FPGA is advantageous.

It is important to note that the equations presented in this paper can be easily specified in synthesizable HDL (VHDL or Verilog) code and that the developed convolution mask algorithm is described in detail to support its implementation also in HDL code.

In future work, filters will be optimized to reduce the number of clock cycles and possibly the circuit area. Frame transmission to the CPU can be more efficient, e.g., image reception. Finally, the library will be extended by implementing more methods.

## Figures and Tables

**Table 1:** Registers and buffers used in the deve lopment of modules that use the convolution mask.

Register/Buffer	Description
Resolution_x	Number of pixels in each row of a frame
Resolution_y	Number of pixels in each column of a frame
Count_x	Horizontal position of the current pixel
Count_y	Vertical position of the current pixel
Buffer_1	Contains row y-2 pixel values (y represents the current row)
Buffer_2	Contains row y-1 pixel values
Buffer_3	Contains the values of the two previous pixels

**Table 2:** Processing times for the methods developed on the FPGA.

Developed Methods	Time (ns)	Clock Cycles
RGB/Gray	10	1
RGB/YCbCr	10	1
Inverse	10	1
Brightness	10	1
Binary	10	1
Sobel	60	6
Mean	40	4
Gaussian	30	3
Erosion	10	1
Dilation	10	1

**Table 3:** Processing times for the methods on the GPU.

	Time per Frame (ms)		Time per Pixel (ns)	
**Methods**	**Mean**	**Faster**	**Mean**	**Faster**
RGB/Gray	0.092	0.066	0.044	0.032
RGB/YCbCr	0.091	0.065	0.044	0.031
Inverse	0.094	0.064	0.046	0.031
Brightness	0.090	0.067	0.044	0.032
Binary	0.092	0.068	0.044	0.033
Sobel	0.088	0.066	0.042	0.032
Mean	0.086	0.064	0.041	0.031
Gaussian	0.090	0.064	0.043	0.031
Erosion	0.087	0.065	0.042	0.031
Dilation	0.085	0.063	0.041	0.030

**Table 4:** Processing times for the methods on the CPU.

	Time per Frame (ms)		Time per Pixel (ns)	
**Methods**	**Mean**	**Faster**	**Mean**	**Faster**
RGB/Gray	13.87	10.99	6.69	5.30
RGB/YCbCr	19.70	18.99	9.50	9.16
Inverse	7.46	7.01	3.60	3.38
Brightness	17.23	16.01	8.31	7.72
Binary	5.37	5.00	2.59	2.41
Sobel	101.19	79.00	48.80	38.10
Mean	83.67	80.99	40.35	39.06
Gaussian	54.81	53.00	26.43	25.56
Erosion	11.07	9.99	5.34	4.82
Dilation	18.48	18.00	8.91	8.68

**Table 5:** FPGA resource usage.

Developed Methods	LUT (Units)	LUTRAM (Units)	FF (Units)	BRAM (Units)
RGB/Gray	15	0	4	0
RGB/YCbCr	145	0	28	0
Inverse	26	0	27	0
Brightness	29	0	28	0
Binary	2	0	27	0
Sobel	3052	1920	547	4.5
Mean	4087	2880	336	4.5
Gaussian	4205	2880	367	4.5
Erosion	2951	2160	168	0
Dilation	2972	2160	154	0

**Table 6:** Processing times of the filter sequen ce used on different platforms (FPGA, GPU, CPU).

Platform	Time per Pixel (ns)	Time per Frame (ms)
FPGA	80	-
Equations used on FPGA (CPU)	55.6109	115.3149
Equations used on FPGA (GPU)	0.1667	0.3458
Halcon (CPU)	14.5828	30.2389
OpenCV (CPU)	17.9598	37.2415

**Table 7:** Power consumption of the three use d platforms (FPGA, CPU, and GPU) in Watts.

Platform	Power Consumption (W)
FPGA	1–5
CPU	15
GPU	10–125

## Data Availability

The original contributions presented in the study are included in the article, further inquiries can be directed to the corresponding author.
